# Mechanisms of the Penetration of Blood-Borne Substances into the Brain

**DOI:** 10.2174/157015909788848901

**Published:** 2009-06

**Authors:** Masaki Ueno

**Affiliations:** Department of Pathology and Host Defense, Faculty of Medicine, Kagawa University, Kagawa 761-0793, Japan

**Keywords:** Blood-brain barrier, P-glycoprotein, tight junction.

## Abstract

The blood-brain barrier (BBB) impedes the influx of intravascular compounds from the blood to the brain. Few blood-borne macromolecules are transferred into the brain because vesicular transcytosis in the endothelial cells is considerably limited and the tight junction is located between the endothelial cells. At the first line of the BBB, the endothelial glycocalyx which is a negatively charged, surface coat of proteoglycans, and adsorbed plasma proteins, contributes to the vasculoprotective effects of the vessels wall and are involved in maintaining vascular permeability. In the endothelial cytoplasm of cerebral capillaries, there is an asymmetrical array of metabolic enzymes such as alkaline phosphatase, acid phosphatase, 5’-nucleotidase, adenosine triphosphatase, and nucleoside diphosphatase and these enzymes contribute to inactivation of substrates. In addition, there are several types of influx or efflux transporters at the BBB, such as P-glycoprotein (P-gp), multidrug resistance associated protein, breast cancer resistance protein, organic anion transporters, organic cation transporters, organic cation transporter novel type transporters, and monocarboxylic acid transporters. P-gp, energy-dependent efflux transporter protein, is instrumental to the barrier function. Several findings recently reported indicate that endothelial P-gp contributes to efflux of undesirable substances such as β-amyloid protein from the brain or periarterial interstitial fluid, while P-gp likely plays a crucial role in the genesis of multiple vascular abnormalities that accompany hypertension. In this review, influx and efflux mechanisms of drugs at the BBB are also reviewed and how medicines pass the BBB to reach the brain parenchyma is discussed.

## 1. INTRODUCTION

**Fig. (1):**
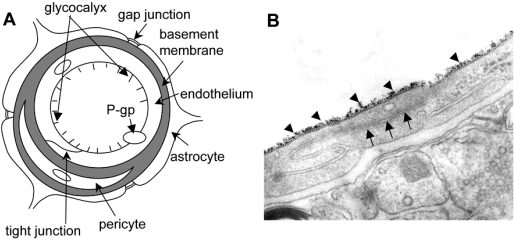
Blood-brain barrier and the tight junction. (**A**) Schematic outline of a capillary of the blood-brain barrier in a transverse section showing the endothelium with a tight junction, basement membrane, a pericyte, and astrocytes. The localization of a gap junction between end-feet of the astrocytes is conceptually shown. (**B**) An electron micrograph showing a typical mammalian endothelial cell combined with a tight junction (arrows) and cationized ferritin (arrowheads) bound to the luminal endothelial surface representing the endothelial glycocalyx. There are scarce vesicular structures in the typical ECs of the brain.

The mammalian brain restricts the entrance of ions and solutes circulating in the bloodstream by the blood-brain barrier (BBB) [13]. The BBB is built up by a monolayer of endothelial cells (ECs) lining the brain capillaries that restricts the movement of small polar molecules and macromolecules between the blood and the brain interstitial fluid with tight junctions between ECs [12,65,88]. The endothelial barrier is supplemented with capillary pericytes that share the basement membrane with the ECs. Moreover, the astrocyte perivascular end-feet almost totally cover the abluminal surface of the microvascular basement membrane (Fig. **1**).

The brain capillaries were characterized morphologically as the site of the BBB by Reese and Karnovsky [65] after introduction of electron microscopy and the use of horseradish peroxidase as a macromolecular tracer. Further ultrastructural studies revealed that the continuous endothelium of brain capillaries possesses several unique structural and functional features [11,85,86,89].

In this review, not only ultrastructural features at the BBB but also functional expression of several transporters such as P-gp are discussed. Information on transportation of blood-borne substances from the bloodstream to the brain parenchyma will be useful to understand how medicines are taken into the brain.

## 2. MOLECULAR ANATOMY IN THE BRAIN ENDOTHELIAL CELL BARRIER

### 2.1. Tight Junctions (TJ)

Junctional complexes in the ECs of cerebral capillaries are comprised of TJ and adherens junctions (AJ) (Fig. **1**). The TJs ultrastructurally appear as sites of apparent fusion involving the outer leaflets of the plasma membrane [14, 41,54]. The TJ consists of three integral membrane proteins, namely, claudin, occludin, and junction adhesion molecules, and a number of cytoplasmic accessory proteins including zonula occludens (ZO)-1, ZO-2, ZO-3, cingulin, and others [20,33,34]. AJs are composed of a cadherin-catenin complex and its associated proteins. Cytoplasmic proteins link membrane proteins to actin, which is the primary cytoskeleton protein in the maintenance of the structural and functional integrity of the endothelium.

### 2.2. Molecules in the Transcytotic Pathway for Blood-Borne Proteins

Non-lipid-soluble micromolecules and macromolecules are capable of circumventing the “fluid-brain barrier” by intracellular routes related to three separate and distinct endocytic processes [15,16], namely, (a) fluid-phase endocytosis, (b) adsorptive endocytosis, and (c) receptor-mediated endocytosis. (a) Fluid-phase endocytosis is a constitutive process for acquiring extracellular macromolecules and recycling of the plasma membrane. This internalization process occurs indiscriminately and without binding to the cell surface [16]. (b) Adsorptive endocytosis concerns molecules such as lectins that bind to carbohydrate moieties on the cell surface (e.g., wheat germ agglutinin), and positively charged (cationized) molecules that bind to negatively charged cell surface components. (c) Receptor-mediated endocytosis has been identified in clathrin-coated vesicles with the binding of a ligand (e.g., insulin, transferrin) to a cell surface receptor specific for that ligand; the binding then triggers the internalization of the receptor-ligand complex. Clathrin-mediated endocytosis from the plasma membrane allows cells to internalize proteins and other biomolecules from their environment *via* specific receptors. Receptors are endocytosed by their capture in clathrin-coated vesicles budding from the plasma membrane. In addition, vesiculo-vacuolar organelle [42] and vesiculo-tubular structures [51,77] have been suggested as transendothelial pathways for macromolecular extravasation.

## 3. ULTRASTRUCTURAL CYTOCHEMISTRY OF ENZYMATIC AND NON-ENZYMATIC CONSTITUENTS OF BRAIN ENDOTHELIA

### 3.1. Enzymatic Activity

Distribution patterns of several activity detected by enzymatic histochemistry in fixed samples have been reported.

#### (a) Alkaline Phosphatase (ALPase)

It is well known that ALPase is a nonspecific enzyme and can hydrolyze various phosphate esters both organic and inorganic, like α-naphthyl phosphate, p-nitrophenyl phosphate, AS-naphthols, etc. In the majority of animal species including mammals, the ECs of the brain microvessels of BBB type show a high acivity of ALPase. The ultrastructural localization of the reaction product for ALPase activity is different in brain as compared to muscle capillary endothelia [90]. In capillary ECs of mice, the positive reaction appears on the blood-oriented surface of the luminal plasma membrane. On the contrast, in the arterioles, the reaction product is present on both sides (luminal and abluminal) of the ECs [88]. In guinea pig [40], a positive reaction for ALPase appears on both luminal and abluminal fronts of the ECs in brain capillaries. The localization of the ALPase is changed in pathological conditions induced by chronic relapsing experimental allergic encephalomyelitis.

#### (b) Acid Phosphatase (ACPase)

ACPase is a typical lysosomal enzyme considered to be an excellent enzymatic marker of these organelles [60] and is negligible in normal brain ECs. It can play an important emergency role in pathological and abnormal conditions as one of the digestive enzymes of the lysosomal system present in great abundance in perivascular cells.

#### (c) 5’-Nucleotidase (5’-N)

It is a generally accepted view that 5’-N is a typical plasmalemmal enzyme, and its activity has been used as a marker of purity of the isolated plasma membrane fractions [9]. 5’-N is transmembrane glycoprotein with sugar residues and the transmembrane location of the enzyme is typical for transport enzymes with hydrolytic sites. Because phosphorylytic cleavage of adenosine-5’-phosphate is necessary for the uptake of adenosine and for facilitation of its transport across the cell membrane [6], the importance of 5’-N in this processes is generally acknowledged.

#### (d) Adenosine Triphosphatase - Na^+^,K^+^-ATPase –

The enzymatic activity of ATPase appears mainly in the cell membranes. It is very sensitive to aldehyde fixatives and accordingly controversial findings have been reported. In some cases, the ATPase is activated by Mg^2+^, in some by Ca^2+^, and in other cases by both Ca^2+^ and Mg^2+^. Na^+^,K^+^- ATPase is the ouabain-sensitive sodium pomp that concentrates potassium in the cytoplasm and deplete intracellular sodium. The results of several observations indicated a distribution pattern of the enzymatic activity of Na^+^, K^+^- ATPase, mainly on the abluminal side of the ECs of the rat brain capillary was observed by Firth [28] and by Betz et al. [7], while other authors [38,39] did not find a positive reaction for ATPase in the rat brain capillaries after incubation in a Mayahara medium. It is likely that the enzymatic activity of Na^+^, K^+^-ATPase present in the wall of brain microvessels, especially in the membranes adjacent to the basement membrane. Thus, Na^+^, K^+^-ATPase would be responsible for the maintenance of the optimal level of both cations in cells and in interstitial fluids.

#### (e) Nucleoside Diphosphatase (NDPase)

The activity of NDPase is commonly used as one of the marker enzymes for the demonstration of the endoplasmic reticulum (ER) in several types of cells [60]. In the mouse central nervous system, the reaction product appeared in neurons, astrocytes and oligodendrocytes in the cisternae of the ER or only in the trans-elements of the Golgi apparatus [88].

### 3.2. Glycocalyx

The glycocalyx is a negatively charged, surface coat of proteoglycans, glycosaminoglycans, and adsorbed plasma proteins lining the luminal surface of the endothelium [52]. Some researchers have put forward the concept that the endothelial glycocalyx contributes to the vasculoprotective effects of the vessel wall [59]. This layer has also been shown to be involved in maintaining vascular permeability [35]. The endothelial glycocalyx can be evaluated by measuring the binding capacity of cationized ferritin on the luminal endothelial surface [83] (Fig. **1B**). In addition, the glycocalyx harbours a wide array of enzymes that might contribute to its vasculoprotective effect. Extracellular superoxide dismutase, an enzyme that converts oxygen radicals to hydrogen peroxide, is bound to heparan sulphate proteoglycans within glycocalyx [47]. Damage to the glycocalyx is accompanied by increased shedding of extracellular superoxide dismutase, which is probably related to the decreased availability of heparan sulphate binding sites. The glycocalyx damage shifts the balance towards a pro-oxidant state. These observations are of particular interest because altered vascular permeability, attenuated nitric oxide bioavailability, and redox dysregulation are the earliest characteristics of atherogenesis [48]. In addition, a disappearance of the glycocalyx is expected to be followed by the exposure of adhesion molecules on ECs and subsequent leukocyte rolling, tethering and transmigration, which are critical in the course of atherogenesis [48]. This evidence suggests that the intact glycocalyx is necessary for the maintenance of normal vascular function, and that disruption of glycocalyx by atherogenic stimuli increases the vascular vulnerability to atherogenesis. Moreover, it is known that endothelial glycocalyx is disturbed in various types of vascular diseases [59]. It is also known that inflammation induces glycocalyx shedding [55]. One of the most common chemokines expressed in the CNS during inflammation is monocyte chemoattractant protein-1 [25]. High chemokine expression is found in many pathological settings accompanied by inflammation, providing a chemoattractant gradient for leukocyte influx to the brain [56,67].

## 4. TRANSPORTERS IN BRAIN MICROVASCULATURE

The blood-to-brain influx transporters supply hydrophilic nutrients and other essential molecules such as glucose [63], lactate/monocarboxylates [22], and creatine [61]. In addition, L-tyrosine, L-tryptophan, and L-histidine are precursors of neurotransmitters, and are transported from the blood to the brain *via* a Na^+^-independent neutral amino acid transporter (system L) at the BBB [62]. The system L is potentially important for drug delivery to the brain. L-Dopa is transported across the BBB by system L, and is ready biotransformed in the brain to dopamine [32]. On the other hand, there are several kinds of efflux transporters at the BBB such as ATP-binding cassette (ABC) transporters, organic anion transport (OAT) systems, aminoacid transport systems, and so on [62].

Major representatives of the ABC efflux transporters are the multidrug resistance protein (MDR), multidrug resistance associated protein (MRP), and breast cancer resistance protein [80]. P-glycoprotein (P-gp), which belongs to the MDR family, preferentially transports cationic and /or zwitterionic compounds as substrates, whereas the MRP family preferentially transports anionic compounds, although there is some overlap between them [36]. Newly discovered categories of transporters are the OAT family, the organic cation transporter (OCT) family, the organic cation transporter novel type (OCTN)/carnitine transporter family [1,74], and the monocarboxylic acid transporter (MCT) family [64], which is expected to be responsible for the transport of some organic anions from the brain to the EC and/or from the EC to the blood [80]. In addition, the concentrative nucleoside transporter and equilibrative nucleoside transporter subfamilies have also detected in brain capillaries or brain capillary EC lines [3].

### (a) P-gp

#### <i> Function and Distribution of P-gp

**Fig. (2):**
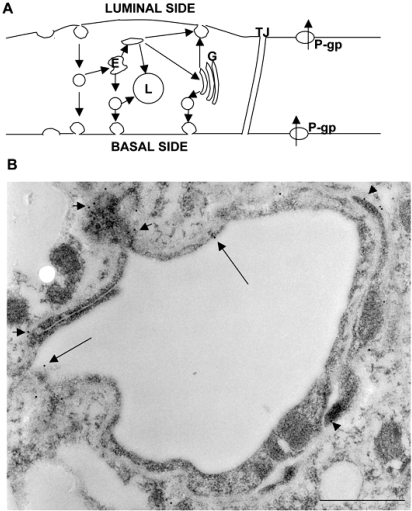
(**A**) Hypothetical transcytotic routes through ECs of the BBB and membrane events associated with endocytic processes are represented. Direct transendothelial vesicular transport or an indirect route through the endosomal compartment (**E**) may apply to the receptor-mediated transcytosis of blood-borne ligands. Some endocytic vesicles derived from the luminal surface membranes are likely directed to endosomes that give rise to exocytic vesicles. Macromolecules entering the endothelium by adsorptive endocytosis are channeled to endosomes and either direcly or indirectly to the inner saccule of the Golgi complex (**G**); this Golgi saccule is responsible for packaging macromolecules for exocytosis at the abluminal and luminal faces of the endothelium. Fluid-phase macromolecules and some macromolecules taken into the endothelium by receptor-mediated and adsorptive endocytic processes are directed to endosomes (**E**) and lysosomes (**L**). The macromolecules deposited in lysosomes are enzymatically degraded rather than transcytosed. The multidrug resistance efflux transporter P-gp is shown at the luminal and abluminal membranes of the endothelial cytoplasm. (**B**) A rat brain was removed after perfusion with physiological saline and immersion-fixed with 4% paraformaldehyde in 0.1M phosphate buffer (PB). The brain tissue was embedded in LR White resin after additional fixation in 1% glutaraldehyde in 0.1M PB for 1 hour. Ultrathin sections were stained with anti-P-gp antibody, followed by incubation in a solution of anti-mouse IgG antibody conjugated with colloidal gold particles of 10 nm diameter (EY Laboratories, CA), diluted with phosphate buffered saline (1:25), for 1 h at RT. The ultrathin secions were stained with uranyl acetate and Reynold’s lead citrate, and were examined in a JEM-1200EX electron microcope (JEM, Tokyo, Japan). Labeling by 10-nm gold particles conjugated with the antibody against P-gp is found in the cytoplasm including the luminal (long arrows) and abluminal (short arrows) membranes of the ECs, and the basal lamina. The scale bar indicates 500 nm.

The multidrug resistance efflux transporter P-gp was the plasma membrane protein first demonstrated in cancer cells by reducing intracellular levels of chemotherapeutic drugs [49]. However, P-gp is also expressed in various normal tissues such as the liver, kidney, intestine, and brain, where it functions to protect the tissue against potentially toxic exogenous compounds [8,29,69]. In addition, it is known that P-gp is identified not only in normal epithelial cells with secretory/excretory functions but also in the ECs of the capillary blood vessels in the brain [70] and the testis [53]. Until quite recently, P-gp in the brain had been thought to be primarily located in the apical (luminal) membrane of capillary ECs that form the BBB and to become part of the mechanisms involved in protecting the brain from xenobiotics [4,23,68]. A recent study using a new polyclonal antibody against P-gp [71] demonstrated dual expression of P-gp at astrocytes and the endothelium in normal primate brains. In addition, Bendayan *et al*. [5] recently reported that P-gp localized to both the luminal and abluminal membranes of capillary ECs, as well as in adjacent pericytes and astrocytes (Fig. **2**). These authors reported that P-gp was distributed along the nuclear envelope, in the caveolae, cytoplasmic vesicles, Golgi complex, and rough endoplasmic reticulum. They stated that this glycoprotein might regulate drug transport processes in the CNS at both the cellular and subcellular levels.

#### <ii> P-gp Related Drug-Drug Interaction

P-gp substrates include not only a wide variety of antineoplastic agents, but also many other hydrophobic compounds, such as immunosuppressive agents, cardiac glycosides, opioid analgesics, antibiotics, pesticides, antiepileptics, antidepressants, and human immunodeficiency virus protease inhibitors [69]. Inhibition of P-gp can be achieved by antidepressants [91], suggesting the possibility that using a medicine together with an antidepressant may lead to an increase in the brain concentration of the medicine. It is also shown that at clinically relevant doses given orally, oxytetracycline is able to saturate P-gp and, subsequently, the net absorption of other drugs increases [72]. In addition, the large number of psychoactive drugs that are substrates of P-gp could be potentially involved in s significant number of drug-drug interactions regarding P-gp. Because of overlapping substrates specificities between CYP3A4 and P-gp, many drug interactions may involved both CYP2A4 and P-gp [50]. Therefore, it is important to distinguish CYP3A4-mediated from P-gp-mediated inhibition in order to make appropriate interpretation of drug interaction data.

#### <iii> P-gp and Neurodegenerative Disease

P-gp deficiency induces an undesirable effect on the brain. It has been hypothesized that Aβ proteins are deposited in periarterial interstitial fluid drainage pathways of the brain, contributing significantly to cerebral amyloid angiopathy in Alzheimer’s disease [92]. Vogelgesang *et al*. [87] reported that Aβ deposition occurred first in arterioles, where P-gp expression was primarily low, and disappeared completely with the accumulation of Aβ proteins. In addition, Cirrito *et al*. [19] reported that P-gp deficiency at the BBB increased amyloid-β deposition in a murine model of Alzheimer’s disease, suggesting that P-gp normally discharges Aβ out of the brain or periarterial interstitial fluid, and that perturbation of Aβ efflux directly affects Aβ accumulation within the brain or perivascular areas. It was very recently reported that P-gp expression was increased in the BBB-damaged vessels of a stroke-prone hypertensive rat [84]. It seems to be likely that the expression of P-glycoprotein increases as a temporary physiological compensatory response in BBB-damaged vessels to discharge intracerebral or periarterial undesirable substances from the brain. These findings suggest that endothelial P-gp contributes to efflux of undesirable substances from the brain or periarterial interstitial fluid. Concerning transendothelial transport of β-amyloid protein, the receptor for advanced glycation end products (RAGE) is thought to be a primary transporter of β-amyloid across the BBB into the brain from systemic circulation, while the low-density lipoprotein receptor-related protein (LRP)-1 mediates transport of β-amyloid out of the brain [26,94]. RAGE versus LRP balance regulates Alzheimer amyloid β-peptide clearance through transport across the BBB [24]. Accordingly, it is reasonable to think that plural factors contribute to removal of β-amyloid. In addition, BBB efflux function of the P-gp transport system was decreased at later disease stages of Parkinson’s disease, suggesting that the P-gp dysfunction contributes to neuronal damage due to increased accumulation of toxins, such as insoluble α-synuclein [2].

#### <iiii> P-gp and Vascular Abnormalities

According to a paper reported by Widder *et al.* [93], the P-gp is a major exporter of oxidized glutathione, and plays a crucial role in the genesis of multiple vascular abnormalities that accompany hypertension. Moreover, its presence is essential for the hypertensive response to angiotension II. The findings suggest that the increased expression of P-gp in BBB-damage vessels may induce directly the BBB damage.

### (b) MRP

MRP1 is a member of the ATP-binding cassette superfamily and is expressed in non-P-gp expressing MDR cell lines [21]. Of the MRP family, MRP1, MRP3, and MRP5 are expressed in the BBB [37,43,66]. Since MRP is involved in extrusion of conjugated xenobiotics that may be harmful to the brain, some authors suggest that MRP1 and/or its closely related proteins are expressed at the luminal side of the brain capillaries [45,73]. However, this has not been proven experimentally.

### (c) OAT Family

OATs are multispecific organic anion transporters, the substrates of which include both endogenous (e.g. cyclic nucleotides, prostaglandins, urate) and exogenous anions. OAT1 and OAT3 were demonstrated to be present on the brushborder of the choroid plexus [76] and in an *in vitro* BBB model of cultured mouse brain ECs [44], respectively. Various organic anions are proposed as substrates of OAT, such as p-aminohippurate, dicarboxylates, and β-lactam antibiotics.

### (d) OCT Family

OCTs transport a variety of cationic compounds, including monoamine neurotransmitters and classical organic cation transporter substrates such as tetraethylammonium, and choline. Rat OCT3 and human OCT2 are expressed in the brain. It is thought that they participate in the regulation of neurotransmitters in neurons rather than at the BBB [80]. 

A new family of OCTNs has been also found [81]. The amino acid sequences of OCTNs show low, but significant, similarity to those of OCT and OAT family members. The OCTNs were initially thought to be organic cation transporters. However, subsequent studies clarified that they physiologically functioned as sodium ion-dependent transporters for carnitine, which is important in the metabolism of fatty acids [58,79].

### (e) MCT Family

It is known that MCT1 mediates the transport of lactic acid and pyruvic acid [30]. The MCT1 gene was detected in rat brain capillaries by using a RT-PCR method [78]. The MCT1 protein was immunohistochemically detected at both luminal and abluminal membranes of the brain capillary ECs [31]. It is thought that the MCT1 at the BBB contributes to the regulation of energy substrates in the brain parenchyma.

## 5. POTENTIAL PATHWAY OF BLOOD-BORNE COMPOUNDS INTO THE BRAIN

Mentioned above, the endothelial glycocalyx with extracellular enzymes covers the luminal surface of the ECs and accordingly works at the first line of the BBB. The ECs of brain capillaries are morphologically characterized with limited vesicular transcytosis and tight junctions. Enzymatic constituents in the endothelial cytoplasm of brain capillaries inactivate some substrates. The endothelial transcytosis in brain capillaries is limited to specific substrates because several kinds of influx and efflux transporters are located at the BBB. In these ways, the BBB impedes the influx of intravascular compounds from the blood to the brain. In order to work medicines on brain function, medicines should be transferred into the brain through the BBB, and the medicines entering the brain should be escaped from discharge into the blood by the transporters or modification by the enzymes. Various trials for medicines to pass the BBB into the brain parenchyma have been performed.

Osmotic opening of TJs has been reported in several types of animal models [57], since the original reports by Broman and Olsson in the 1940s [18]. It is likely that reversible opening of TJs would be useful for delivery of medicines into the brain. It is also known that the intra-arterial administration of alkylglycerols transiently increases the penetration of drugs and macromolecules across the BBB, suggesting that the administration of alkylglycerols could be a unique method for enhanced drug delivery to the brain and to brain tumors [27,46]. It is most likely that increased lipophilicity of drugs makes transportation into the brain easy. Compounds bound to lectins are thought to be easily transported by adsorptive vesicular transport. Enhanced vesicular transport can be used to deliver compounds into the brain. Transient inhibition of P-gp by medicines such as antidepressants may be useful for delivery of anti-cancer drugs into the brain. It is likely that the manipulation of P-gp will be useful for delivery of medicines in brain and cerebrovascular diseases. If a targeted region is situated near one of the circumventricular organs (CVOs), the delivery of medicines to the region could be achieved *via* CVO capillaries. There are extracellular pathways bypassing the BBB. Blood-borne proteins gaining extracellular access to non-BBB sites can move not only within the cerebrospinal fluid of the subarachnoid space, but also into the brain parenchyma adjacent to each of the leaky sites [17]. It is possible in experimental animals that blood-borne macromolecules escaping the subfornical organ, a BBB-free area, have ready access not only to the white matter of the corpus callosum [17], but also to the hippocampus [82]. A drainage pathway through the subarachnoid spaces of olfactory nerves from the brain to deep cervical lymph nodes has been also proposed by Bradbury *et al*. [10]. Accordingly, nasally inhaled medicines can affect parts of the brain through the subarachnoid spaces of olfactory nerves.

In addition, it has been investigated in experimental animals whether the treatment of brain diseases is possible by using gene targeting technology that delivers the gene across the BBB after i.v. administration of nonviral formulation of the gene [75]. In the experiment, the plasmid DNA was targeted to brain with pegylated immunoliposomes using a targeting ligand such as an antibody to transferrin receptor or insulin receptor.

Thus, detailed information on the BBB is necessary and useful to plan a strategy and develop therapies against various brain and vascular diseases.

## ABBREVIATIONS

ABC: = ATP-binding cassette
ACPase: = Acid phosphatase
ALPase: = Alkaline phosphatase
AJ: = Adherens junction
ATPase: = Adenosine triphosphatase
BBB: = Blood-brain barrier
CNS: = Central nervous system
CSF: = Cerebrospinal fluid
CVO: = Circumventricular organ
EC: = Endothelial cell
ER: = Endoplasmic reticulum
MCT: = Monocarboxylic acid transporter
MDR: = Multidrug resistance protein
MRP: = Multidrug resistance associated protein
5’-N: = 5’-Nucleotidase
NDPase: = Nucleoside diphosphatase
OAT: = Organic anion transporter
OCT: = Organic cation transporter
OCTN: = Organic cation transporter novel type
P-gp: = P-glycoprotein
TJ: = Tight junction
ZO: = Zonula occludens

## References

[1] Bart J, Groen HJM, Hendrikse NH, van der Graaf WTA, Vaalburg W, de Vries EGE (2000). The blood-brain barrier and oncology: new insights into function and modulation. Cancer Treat. Rev.

[2] Bartels AL, Willemsen ATM, Kortekaas R, de Jong BM, de Vries R, de Klerk O, van Oostrom JCH, Portman A, Leenders KL (2008). Decreased blood-brain barrier P-glycoprotein function in the progression of Parkinson’s disease, PSP and MSA. J. Neural. Transm.

[3] Bauer B, Hartz AWS, Fricker G, Miller DS (2005). Modulation of P-glycoprotein transport function at the blood-brain barrier. Exp. Biol Med.

[4] Bendayan R, Lee G, Bendayan M (2002). Functional expression and localization of P-glycoprotein at the blood brain barrier. Microsc Res Tech.

[5] Bendayan R, Ronaldson PT, Gingras D, Bandayan M (2006). *In situ* localization of P-glycoprotein (ABCB1) in human and rat brain. J. Histochem. Cytochem.

[6] Berman JJ, Tong C, Williams GM (1980). 5’-Nucleotidase activities in cultures rat liver epithelial and fibroblast cells. J. Histochem. Cytochem.

[7] Betz AL, Firth TA, Goldstein GW (1980). Polarity of the blood-brain barrier: Distribution of enzymes between the luminal and antiluminal membranes of brain capillary endothelial cells. Brain Res.

[8] Bodo A, Bakos E, Szeri F, Varadi A, Sarkadi B (2003). The role of  multidrug transporters in drug availability, metabolism and toxicity. Toxicol. Lett.

[9] Bosmann HB, Pike GZ (1971). Membrane marker enzymes: Isolation, purification and properties of 5’-nucleotidase from rat cerebellum. Biochim. Biophys. Acta.

[10] Bradbury MWB, Cserr HF, Westrop RJ (1981). Drainage of cerebral interstitial fluid into deep cervical lymph of the rabbit. Am. J. Physiol.

[11] Brightman MW, Neuwelt E.A (1981). The anatomic basis of the blood-brain barrier. Implications of the Blood-Brain Barrier and Its Manipulation.

[12] Brightman MW, Reese TS (1967). Junctions between intimately apposed cell membranes in the vertebrate brain. J. Cell Biol.

[13] Brightman MW, Reese TS, Feder N, Crone C, Lassen NA (1970). Assessment with the electron microscope of the permeability to peroxidase of cerebral endothelium and epithelium in mice and shrks. Capillary permeability.

[14] Brightman MW, Tao-Cheng JH, Pardridge WM (1993). Tight junctions of brain endothelium and epithelium. The Blood-Brain Barrier.

[15] Broadwell RD (1992). Pathways into, through, and around the fluid-brain barrier. NIDA Res. Monogr.

[16] Broadwell RD, Balin BJ, Salcman M (1988). Transcytotic pathway for blood-borne protein through the blood-brain barrier. Proc. Natl. Acad. Sci. U.S.A.

[17] Broadwell RD, Sofroniew MV (1993). Serum proteins bypass the blood-brain fluid barriers for extracellular entry to the central nervous system. Exp. Neurol.

[18] Broman T, Olsson O (1948). The tolerance of cerebral blood vessels to a constrast medium of the diodrast group. Acta Radiol. (Stockh).

[19] Cirrito JR, Deane R, Fagan AM, Sprinner ML, Parsadanian M, Finn MB, Jiang H, Prior JL, Sagare A, Bales KR, Paul SM, Zlokovic BV, Piwnica-Worms D, Hotzman DM (2005). P-glycoprotein deficiency at the blood-brain barrier increases amyloid-β deposition in an Alzheimer disease mouse model. J. Clin. Invest.

[20] Citi S (1993). The molecular organization of tight junctions. J. Cell Biol.

[21] Cole SP, Bhardwaj G, Gerlach JH, Mackie JE, Grant CE, Almquist KC, Stewart AJ, Kurz EU, Duncan AM, Deeley RG (1992). Overexpression of a transporter gene in a multidrug-resistant human lung cancer cell line. Science.

[22] Cremer JE, Cunningham VJ, Pardridge WM, Braun LD, Oldendorf WH (1979). Kinetics of blood-brain barrier transport of pyruvate, lactate and glucose in sucking, weanling and adult rats. J. Neurochem.

[23] de Boer AG, van der Sandt IC, Gaillard PJ (2003). The role of drug transporters at the blood-brain barrier. Annu. Rev. Pharmacol. Toxicol.

[24] Deane R, Wu Z, Zlokovic BV (2004). RAGE (Yin) versus LRP (Yang) balance regulates Alzheimer amyloid β-peptide clearance through transport across the blood-brain barrier. Stroke.

[25] Dimitrijevic OB, Stamatovic SM, Keep RF, Andjelkovic AV (2006). Effects of the chemokine CCL2 on blood-brain barrier permeability during ischemia-reperfusion injury. J. Cereb. Blood Flow Metab.

[26] Donahue JE, Flaherty SL, Johanson CE, Duncan III JA, Silverberg GD, Miller MC, Tavares R, Yang W, Wu Q, Sabo E, Hovanesian V, Stopa EG (2006). RAGE, LRP-1, and amyloid-beta protein in Alzheimer’s disease. Acta Neuropathol.

[27] Erdlenbruch B, Jendrossek V, Eibl H, Lakomek M (2000). Transient and controllable opening of the blood-brain barrier to cytostatic and antibiotic agents by alkylglycerols in rats. Exp. Brain Res.

[28] Firth JA (1977). Cytochemical localization of the K^+^ regulation interface between blood and brain. Experientia.

[29] Fromm MF (2003). Importance of P-glycoprotein for drug disposition in humans. Eur. J. Clin. Invest.

[30] Garcia CK, Goldstein JL, Pathak RK, Anderson RGW, Brown MS (1994). Molecular characterization of a membrane transporter for lactate, pyruvate, and other monocarboxylates: implications for the Cori cycle. Cell.

[31] Gerhart DZ, Enerson BE, Zhdankina OY, Leino RL, Drewes LR (1997). Expression of monocarboxylate transporter MCT1 by brain endothelium and glia in adult and sucking rats. Am. J. Physiol.

[32] Gomes P, Soares-da-Silva P (1999). L-Dopa transport properties in an immortalized cell line of rat capillary cerebral endothelial cells. RBE4. Brain Res.

[33] Gumbiner B, Lowenkopf T, Apatira D (1991). Identification of a 160-kDa polypeptide that binds to the tight junction protein ZO-1. Proc. Natl. Acad. Sci. USA.

[34] Haskins J, Gu L, Wiichen ES, Hibbard J, Stevenson BR (1998). ZO-3, a novel member of the MAGUK protein family found at the tight junction, interacts with ZO-1 and occludin. J. Cell Biol.

[35] Henry CB, Duling BR (1999). Permeation of the luminal capillary glycocalyx is determined by hyaluronan. Am. J. Physiol.

[36] Hollo Z, Homolya L, Hegedus T, Sarkadi B (1996). Transport properties of the multidrug resistance-associated protein (MRP) in human tumor cells. FEBS Lett.

[37] Huai-Yun H, Secrest DT, Mark KS, Carnev D, Braudquist C, Elmquist WF, Miller DW (1998). Expression of multidrug resistance-associated protein (MRP) in brain microvessel endothelial cells. Biochem. Biophys. Res. Commun.

[38] Inagaki C, Oda W, Kondo K, Kusumi M (1987). Histochemical demonstration of Cl-ATPase in rat soinal motoneurons. Brain Res.

[39] Inomata K, Yoshioka T, Nasu F, Mayahara H (1984). Histochemical studies of capillary endothelial cells in the rat central nervous system. Acta Anat.

[40] Kato S, Nakamura H (1987). Ultrastructural localization of alkaline phosphatase activity in endothelial cells in chronic relapsing experimental allergic encephalomyelitis. Acta Neuropathol.

[41] Kniesel U, Wolburg H (2000). Tight junctions of the blood-brain barrier. Cell. Mol. Neurobiol.

[42] Kohn S, Nagy JA, Dvorak HF, Dvorak AM (1992). Pathways of macromolecular tracer transport across venules and small veins. Structural basis for the hyperpermeability of tumor blood vessels. Lab. Invest.

[43] Kool M, de Haas M, Scheffer GL, Scheper RJ, van Ejik MJ, Juijn JA, Baas F, Borst P (1997). Analysis of expression of cMOAT (MRP2), MRP3, MRP4, and MRP5, homologues of the multidrug resistance-associated protein gene (MRP1), in human cancer cell lines. Cancer Res.

[44] Kusuhara H, Sekine T, Utsunomiya-Tate N, Tsuda M, Kojima R, Cha SH, Sugiyama Y, Kanai Y, Endou H (1999). Molecular cloning and characterization of a new multispecific organic anion transporter from rat brain. J. Biol. Chem.

[45] Kusuhara H, Suzuki H, Naito M, Tsuruo T, Sugiyama Y (1998). Characterization of efflux transport of organic anions in mouse brain capillary endothelial cells. J. Pharmacol. Exp. Ther.

[46] Lee HJ, Zhang Y, Pardridge WM (2002). Blood-brain barrier distruption following the internal carotid arterial perfusion of alkyl glycerols. J. Drug Target.

[47] Li Q, Bolli R, Qiu Y, Tang XL, Murphree SS, French BA (1998). Gene therapy with extracellular superoxide dismutase attenuates myocardial stunning in conscious rabbits. Circulation.

[48] Libby P (2002). Inflammation in atherosclerosis. Nature.

[49] Ling V (1995). P-glycoprotein: its role in drug resistance. Am. J. Med.

[50] Linnet K, Ejsing TB (2008). A review on the impact of P-glycoprotein on the penetration of drugs into the brain. Focus on psychotropic drugs. Eur Neuropsychopharmacol.

[51] Lossinsky AS, Vorbrodt AW, Wisniewski HM (1983). Ultrastructural studies of vesicular and canalicular transport structures in the injured mammalian blood-brain barrier. Acta Neuropathol.

[52] Luft JH (1966). Fine structures of capillary and endocapillary layer as revealed by ruthenium red. Fed. Proc.

[53] Melaine N, Lienard M-O, Dorval I, Gaoscogne CL, Lejeune H, Jegou B (2002). Multidrug resistance genes and P-glycoprotein in the testis of the rat, mouse, guinea pig, and human. Biol. Reprod.

[54] Mitic LL, Van Itallie CM, Anderson JM (2000). Molecular physiology and pathophysiology of tight junctions. I. Tight junction structure and function: lessons from mutant animals and proteins. Am. J. Physiol. Gastrointest. Liver Physiol.

[55] Mulivor AW, Lipowsky HH (2002). Role of glycocalyx in leukocyte-endothelial cell adhesion. Am. J. Physiol. Heart Circ. Physiol.

[56] Murphy PM (1994). The molecular biology of leukocyte chemoattractant receptors. Annu. Rev. Immunol.

[57] Neuwelt EA, Dahlborg SA, Neuwelt EA (1989). Blood-brain barrier disruption in the treatment of brain tumors: Clinical implications. Implications of the blood-brain barrier and its manipulation.

[58] Nezu J, Tamai I, Oku A, Ohashi R, Yabuuchi H, Hashimoto N, Nikaido H, Sai Y, Koizumi A, Shoji Y, Takeda G, Matsuishi T, Yoshino M, Kato H, Ohura T, Tsujimoto G, Hayakawa J, Tsuji A (1999). Primary systemic carnitine deficiency is caused by mutations in a gene encoding sodium ion-dependent carnitine transporter. Nat. Gen.

[59] Nieuwdorp M, Meuwese MC, Vink H, Hoekstra JBL, Kastelen JJP, Stroes ESG (2005). The endothelial glycocalyx: a potential barrier between health and vascular disease. Curr. Opin. Lipidol.

[60] Novikoff AB, Essner E (1962). Pathological changes in cytoplasmic organelles. Fed. Proc.

[61] Ohtsuki S, Tachikawa M, Takanaga H, Shimizu H, Watanabe M, Hosoya K, Terasaki T (2002). The blood-brain barrier creatine transporter is a major pathway for supplying creatine to the brain. J. Cereb. Blood Flow Metab.

[62] Ohtsuki S, Terasaki T (2007). Contribution of carrier-mediated transport systems to the blood-brain barrier as a supporting and protecting interface for the brain; importance for CNS drug discovery and development. Pharm. Res.

[63] Pardridge WM, Oldendorf WH (1975). Kinetics of blood-brain transport of hexoses. Biochim. Biophys. Acta.

[64] Price NT, Jackson VN, Halestrap AP (1998). Cloning and sequencing of four new mammalian monocarboxylate transporter (MCT) homologues confirms the existence of a tranporter family with an ancient past. Biochem. J.

[65] Reese TS, Karnovsky MJ Fine structural localization of a blood-brain barrier to exogenous peroxidase. J. Cell Biol.

[66] Regina A, Koman A, Piciotti M, El Hafny B Center, M.S Bergmann, R Couranud, P.O. Roux F (1998). MRP1 multidrug resistance-associated protein and P-glycoprotein expression in rat brain microvessel endothelial cells. J. Neurochem.

[67] Rollins BJ (1997). Chemokines. Blood.

[68] Schinkel AH (1999). P-glycoprotein a gatekeeper in the blood-brain barrier. Adv. Drug Deliv. Rev.

[69] Schinkel AH, Jonker JW (2003). Mammalian drug efflux transporters of the ATP binding cassette (ABC) family: an overview. Adv. Drug Deliv. Rev.

[70] Schinkel AH, Smit JJ, van Tellingen O, Beijnen JH, Wagennaar EW, van Deemter L, Mol CA, van der Valk MA, Robanus-Maandag EC, Riele HP, Berns AJM, Borst P (1994). Distribution of the mouse mdr1a P-glycoprotein gene leads to a deficiency in the blood-brain barrier and to increased sensitivity to drugs. Cell.

[71] Schlachetzki F, Pardridge WM (2003). P-glycoprotein and caveolin-1 α in endothelium and astrocytes of primate brain. Neuroreport.

[72] Schrickx J, Fink-Gremmels J (2007). P-glycoprotein-mediated transport of oxytetracycline in the Caco-2 cell model. J. Vet. Pharmacol. Therap.

[73] Seetharaman S, Barrand MA, Maskell L, Scheper RJ (1998). Multidrug resistance-related transport proteins in isoklated human brain microvessels and in cells cultures from these isolates. J. Neurochem.

[74] Sekine T, Cha SH, Endou H (2000). The  multispecific organic anion transporter (OAT) family. Pflügers Arch. Eur. J. Physiol.

[75] Shi N, Zhang Y, Boado RJ, Pardridge WM (2001). Brain-specific expression of an exogenous gene after i.v. administration. Proc. Natl. Acad. USA.

[76] Sugiyama Y, Kusuhara H, Suzuki H (1999). Kinetic and biochemical analysis of carrier-mediated efflux of drugs through the blood-brain and blood-cerebrospinal fluid barriers: importance in the drug delivery to the brain. J. Control. Release.

[77] Tagami M, Kubota A, Sunaga T, Fujino H, Mawzawa H, Kihara M, Nara Y, Yamori Y (1983). Increased transendothelial channel transport of cerebral capillary endothelium in stroke-prone SHR. Stroke.

[78] Takanaga H, Tamai I, Inaba S, Sai Y, Higashida H, Yamamoto H, Tsuji A (1995). cDNA cloning and functional characterization of rat intestinal monocarboxylate transporter. Biochem. Biophys. Res. Commun.

[79] Tamai I, Ohashi R, Nezu J, Yabuuchi H, Oku A, Shimane M, Sai Y, Tsuji A (1998). Molecular and functional identification of sodium ion-dependent, high affinity human carnitine transporter OCTN2. J. Biol. Chem.

[80] Tamai I, Tsuji A (2000). Transporter-mediated permeation of drugs across the blood-brain barrier. J. Pharm. Sci.

[81] Tamai I, Yabuuchu H, Nezu J, Sai Y, Oku A, Shimane M Tsuji A (1997). Cloning and characterization of a novel human pH-dependent organic cation transporter, OCTN1. FEBS Lett.

[82] Ueno M, Akiguchi I, Hosokawa M, Yagi H, Takemura M, Kimura J, Takeda T (1994). Accumulation of blood-borne horse-radish peroxidase in medial portions of the mouse hippocampus. Acta Neurol. Scand.

[83] Ueno M, Sakamoto H, Tomimoto H, Akiguchi I, Onodera M, Huang C, Kanenishi K (2004). Blood-brain barrier is impaired in the hippocampus of young adult spontaneously hypertensive rats. Acta Neuropathol.

[84] Ueno M, Nakagawa T, Huang C, Ueki M, Kusaka T, Hosomi N, Kanenishi K, Onodera M, Wu B, Sakamoto H (2009). The expression of P-glycoprotein is increased in vessels with blood-brain barrier impairment in a stroke-prone hypertensive model. Neuropathol. Appl. Neurobiol.

[85] Van Deurs B (1978). Microperoxidase uptake into the rat choroids plexus epithelium. J. Ultrastruct. Res.

[86] Van Deurs B (1980). Structural aspects of brain barriers, with special reference to the permeability of the cerebral endpthelium and choroidal epithelium. Int. Rev. Cytol.

[87] Vogelgesang S, Warzok RW, Cascorbi I, Kunert-Keil C, Schroeder E, Kroemer HK, Siegmund W, Walker LC, Pahnke J (2004). The role of P-glycoprotein in cerebral amyloid angiopathy: implications for the early pathogenesis of Alzheimer’s disease. Curr. Alzheimer Res.

[88] Vorbrodt AW (1988). Ultrastructural cytochemistry of blood-brain barrier endothelia. Prog. Histochem. Cytochem.

[89] Vorbrodt AW, Dobrogowska DH (2003). Molecular anatomy of intercellular junctions in brain endothelial and epithelial barriers: electron microscopist’s view. Brain Res. Rev.

[90] Vorbrodt AW, Lossinsky AS, Wisniewski HM (1986). Localization of alkaline phosphatase activity in endothelial of developing and mature mouse blood-brain barrier. Dev. Neurosci.

[91] Weiss J, Gregor SM, Dormann G, Martin-Facklam M, Kerpen CJ, Ketabi-Kiyanvash N, Haefeli WE (2003). Inhibition of P-glycoprotein by newer antidepressants. J. Pharmacol. Exp. Ther.

[92] Weller RO, Massey A, Newman TA, Hutchings M, Kuo YM, Roher AE (1998). Cerebral amyloid angiopathy: amyloid beta accumulates in putative interstitial fluid drainage pathways in Alzheimer’s disease. Am. J. Pathol.

[93] Widder JD, Guzik TJ, Mueller CFH, Clempus RE, Schmidt HHHW, Dikalov SI, Griendling KK, Jones DP, Harrison DG (2007). Role of the multidrug resistance protein-1 in hypertension and vascular dysfunction caused by angiotensin II. Arterioscler. Thromb. Vasc. Biol.

[94] Zlokovic BV (2005). Neurovascular mechanisms of Alzheimer’s neurodegeneration. Trends Neurosci.

